# Depressive symptomatology, temperament and oxytocin serum levels in a sample of healthy female university students

**DOI:** 10.1186/s40359-022-00744-5

**Published:** 2022-02-22

**Authors:** L. Veiga, E. Carolino, I. Santos, C. Veríssimo, A. Almeida, A. Grilo, M. Brito, M. C. Santos

**Affiliations:** 1 H&TRC-Health and Technology Research Center, ESTeSL- Escola Superior de Tecnologia da Saúde de Lisboa, Instituto Politécnico de Lisboa, Lisbon, Portugal; 2 Medical School – Algarve University, Faro, Portugal; 3 Faculty of Sciences - University of Lisbon, Lisbon, Portugal; 4 Escola Superior de Tecnologia da Saúde de Lisboa/Instituto Politécnico de Lisboa, Av. D. João II, Lote 4.69.01, 1990-096 Lisbon, Portugal; 5 CICPsi - Research Center for Psychological Science, Faculdade de Psicologia, Universidade de Lisboa, Lisbon, Portugal

**Keywords:** Oxytocin levels, Depressive symptomatology, Temperament traits, Female university students

## Abstract

**Background:**

Depressive symptomatology is prevalent among female university students with adverse effects on their quality of life and academic performance. Previous research suggested associations between depressive symptomatology and oxytocin levels and between depressive symptomatology and Temperament Traits. Despite this evidence, to the best of our knowledge no research has studied the effects fboth oxytocin serum levels and temperament dimensions on depressivesymptoms in a healthy sample. The present study aimed to analyse the effect of oxytocin levels and temperament traits on depressive symptomatology in healthy female university students.

**Methods:**

All participants completed the Beck Depression Inventory and the Adult Temperament Questionnaire. Blood samples were collected between 8 and 8H30 a.m. after 12 h of fasting and between 5 and 8 day of the menstrual cycle and serum oxytocin levels were quantified using a commercial enzyme-linked immunosorbent assay. A hierarchical multiple regression model using a stepwise method was conducted to identify predictors of depression.

**Results:**

Forty-five women aged between 18 and 25 years old (19.37 ± 1.32 years) volunteered to participate in this study. Depressive symptomatology was negatively associated with oxytocin serum levels and "Negative affect" and positively associated with "Effortful control" and "Activation Control". In the final regression model, only oxytocin level was a predictor (B = − 0.090, *p* < 0.0001), the model explaining 65.2% of the depression variation. Oxytocin played a mediation role between "Negative affects" and Depressive symptomatology.

**Conclusions:**

Our results showed that oxytocin level, rather than personality dimensions, was associated with depressive symptomatology. These results highlight the relevance of the discussion on the use of oxytocin as a biological marker of emotional and social symptoms that characterize depression.

## Introduction

Depression is a condition marked by sadness, emptiness, feelings of worthlessness, and loss of interest for most of the day activities. Other indicators include insomnia or hypersomnia, fatigue/loss of energy, psychomotor agitation or retardation, inability to concentrate, thoughts of death, and suicide ideation [1].

As stated by the Global Burden of Disease Study [2] depression remains, since 1990, one of the major leading cause of disability globally, reflecting a lack of progress in addressing this condition. A study base on the World Health Organization World Mental Health Surveys in 21 countries (four low/lower-middle income, five upper-middle-income, one lower-middle or upper-middle, and 11 high income), showed that one of the most prevalent individual disorders in university students was Major Depression Disorders (MDD), with rates of 4.5 to 7.7% [3]. Research on the prevalence of depressive symptoms among university students in Europe found rates, for female and male students, respectively, from 12.1 to 45.0%, with lower scores in Western countries, Germany 26.7%/ 22.8%, Denmark 24.9%/12.1%, and higher scores in Eastern countries Poland 45.5%/ 27.3%, and Bulgaria 42.9%/33.8% [4]. Similar results were found in a systematic review [5], with prevalence rates ranging from 10 to 85%, mean prevalence of 30.6%, a rate that exceeds the 21,6% found in the general population. Although epidemiological research shows wide variability in the prevalence of depression among countries, the available data is consistent regarding the higher rates of depression in women when compared to men [4, 6]. Depressive symptoms have a negative impact on the quality of life (QOL) [7] and have been associated with school absenteeism and early school dropout [8, 9], poor concentration, general low capacity for work, and poor academic performance [10, 11].

Oxytocin (OT) a neuropeptide synthesized in the hypothalamus and released by the posterior pituitary in systematic circulation, is knowing, for long, as an important hormone involved in parturition and breastfeeding [10–12]. In addition to peripheral effects, in humans, oxytocin is released by magnocellular excretory cells and binds to the amygdala, striatum, substantia nigra, hypothalamus [12]. This central action seems to be involved in human behaviours.

Existing evidence suggests that oxytocin serum levels play a role in many aspects of social behavior, improving bonding and attachment [13–15] increasing willingness to share emotions [16], positive communication [17] and trust [18, 19] increasing the ability to interpret mental states [20] and modulating the attentional processes [21].

Some of the features inherent to social behavior, such as the openness for relations or stress regulation, are also implicated in other behavioral and psychological processes such as depression, anxiety and post-traumatic stress disorder (PTSD) [22]. Recent research has focused on OT's relevance to developing, maintaining, and treating these disorders.

Research, including clinical trials, have been performed in various conditions, including schizophrenia, anxiety, and depression, and showed an association of this hormone with social individuals' skills, such as bonding, empathy, generosity, and altruism [23].

Mixed findings have been found regarding the correlation between the level of circulating plasma OT and depressive symptomology. Studies with samples of non-human mammals found that higher OT levels either by exogenous administration or by endogenous release, increased positive hedonic states in rats suggesting that OT help alleviate the severity of anhedonia observed in Major Depressive Disorder (MDD) [24]. In humans, low levels of OT have been associated with symptoms that characterize depression, such as loss of interest in maintaining interpersonal relationships [25, 26]. In a population diagnosed with MDD, Scantamburlo et al. [27] found that plasma OT levels were reduced compared to individuals without a mood disorder diagnosis. Similarly, in a study by Gordon et al. with healthy university students, OT was negatively correlated with depressive symptoms, measured by The Beck Depression Inventory (BDI), and psychological distress (i.e. depression and anxiety) proved to be predictors of OT [28]. Reinforcing these results, the study by Ozsoy et al. [29] that compared oxytocin levels between a sample of inpatients with depressive disorder, bipolar affective disorder, depressive episode, and a sample of healthy controls, found that serum oxytocin levels were decreased in patients when compared to controls. These results are consistent with several other studies that showed an inverse association between OT levels and depression [30–32]. However, a recent meta-analysis that include 64 studies with several psychiatric disorders, showed no significant differences in peripheral OT between a healthy adult and the MDD groups [33]. Further, Parker et al. [32] reported that Plasma OT concentration was elevated in depressed subjects compared to healthy controls.

It has been proposed that the oxytocin effect is gender-specific, with OT being more biologically relevant to women. Actually, despite some mixed findings, the inverse correlation between OT and stress and mood disorders has been more consistently found in women [29]. For example, Yuen et al. [34] showed that depressed females exhibited lower OT concentrations than depressed males, irrespective of psychotic status, and, unlike males, depressed females exhibited lower OT concentrations than healthy control females.

More recently, Engel et al. [35] carried out a systematic review and meta-analysis that specifically measured basal endogenous oxytocin concentrations in depressive patients and healthy controls. The assessment of nine studies, included in the meta-analytic procedure, showed non-significant differences in basal endogenous oxytocin concentrations between depressive patients and healthy controls. However, significant heterogeneity in effect was detected, and the authors point out that study designs, hormonal assessments, and clinical and demographic factors could explain these results.

Newly studies have begun to focus on other patient characteristics, e.g. temperament and personality traits, that may contribute to the differing results reported by various studies. Rothbart and Derryberry [36] defined temperament as a constitutionally based individual differences in reactivity and self-regulation in emotional, activational, and attentional processes. This approach identified four central constructs of temperament: (1) Extraversion/ Surgency, that consists of sociability and expressions of pleasure in anticipation of rewards or during high intensity/novel activities and motor activity; (2) Negative affect, that encompasses discomfort, anger/frustration, sadness, fear, and low sociability; (3) Effortful Control, that refers to the ability to voluntarily suppress a predominant response to perform a subdominant response according to environmental demands, detecting errors and planning; and (4) Orienting Sensitivity including perceptual, associative and general Sensitivity to stimulus raising from the environment [37]. It has been understood that temperament regards individual differences that (a) have a strong genetic basis; (b) manifest early in life, and (c) are relatively stable over the lifespan [37, 38].

A large body of research showed evidence of a link between temperament and depressive and anxiety disorders. For instance, in a study with a sample of 4773 subjects, members of the population-base, high levels of Harm avoidance and Pessimism were related to both depressive mood (effect sizes; d = 0.84 and d = 1.25, respectively) and depressive disorder (d = 0.68 and d = 0.68, respectively) [39]. More recently, Katz et al. [40], in a meta-analysis that aimed to quantify the relationships between temperament dimension of Sensitivity, depression and anxiety, found that Punishment sensitivity predicted depression (β = 0.37) and anxiety (β = 0.35), and Reward sensitivity predicted depression (β =  − 0.07).

Despite this evidence, to the best of our knowledge no research has studied the effects of both oxytocin serum levels and temperament dimensions on depressive symptoms in a healthy sample. The current research is part of an extensive study with university students of the Instituto Politécnico de Lisboa (IPL) to evaluate the prevalence of psychological distress, namely anxiety and depression and their correlates. The present study aimed to: i) assess the relationship between serum oxytocin, depression and temperament traits and ii) analyse the effect of serum oxytocin and temperament in depressive symptomatology, in a sample of 45 young, healthy university female students.

## Material and methods

### Participants

The study was announced on the IPL campus during the first semester of classes. Female students (n = 65) who intended to participate contacted the researcher group by email. These students received more detailed information about the study objectives and procedures, including collecting a blood sample to evaluate oxytocin serum levels. Those who maintained the intention to participate (n = 53) were invited to meet the research team in a schedule with four different days/hours. A total of 48 showed at the meeting, but three students did not meet the conditions to participate due to exclusion criteria.

The exclusion criteria included: having a diagnose of psychiatric disorders or major medical disorders, being on medical treatment or taking any pills that interfere with the mood in the last month, being pregnant or lactating.

Finally, a total of 45 students completed a socio-demographic questionnaire, the Beck Depression Inventory (BDI-II) [41] and the Adult Temperament Questionnaire (ATQ) [42] and consented to collect a blood sample.

### Self-report measurers

*The socio-demographic questionnaire* aimed to collect information about the sample's characteristics regarding age, socioeconomic level, and medical report for a chronic illness or psychological disorder.

*The BDI-II inventory* is a widely used instrument to measure depressive symptoms. The 21-item tool measures the level of depressive symptoms on a 3-point scale and demonstrates good reliability. The final score is positively correlated with depression status [41].

We use a Portuguese version of BDI-II [43]. This version results from a study with two populations: college students and a community sample. The Cronbach’s coefficient α was 0.91, and the mean score for college students was 8.88(± 7.85); 7.64(± 7.74) for males and 9.72(± 7.82) for females. A score between 0 and 9 is considered an indicator of Minimal Depression (no depression), a score between 10 and 16 is indicative of Mild Depression, a score between 17 and 29 is a score between 17 and 29 is indicative of a Moderate Depression, a score between 30 and 63 is an indicator of Severe Depression [43].

*The Adult Temperament Questionnaire* (ATQ) is based on Evans and Rothbart [42] Model for Adult Temperament. It is one of the best-established temperament measures and as been used in many studies in several fields of research. The ATQ 7/short is a version of the original ATQ and comprises 77 questions grouped into four-factor scales: Negative Affect; Extroversion, Effortful Control and Guiding Sensitivity.

Negative effect includes four subscales: Fear (i.e., negative affect related to anticipation of distress); Sadness (i.e., negative affect and lowered mood and energy-related to exposure to suffering, disappointment, and object loss); Discomfort. (negative affect related to sensory qualities of stimulation, including intensity, rate or complexity or visual, auditory, smell/taste, and tactile stimulation); and Frustration (i.e., negative affect related to the interruption of ongoing tasks or goal blocking).

Extraversion includes three subscales: Sociability (i.e., the enjoyment derived from social interaction); Positive Affect (i.e., intensity, duration, and frequency of experiencing pleasure; High-Intensity Pleasure (i.e., pleasure related to the situation involving high stimulus intensity, rate, complexity, novelty, and incongruity).

Effortful control includes three subscales: Attentional control (i.e., capacity to focus attention as well as to shift attention when desired); Inhibitory Control (i.e., Capacity to suppress inappropriate approach behaviour; and Activation Control (i.e., Capacity to act when there is a strong tendency to avoid it).

Orienting Sensitivity includes 3 subscales: Neutral Perceptual Sensitivity (i.e., Detection of slight, low intensity stimuli from both with the body and the external environment); Affective Perceptual Sensitivity (i.e., Spontaneous emotionally balanced, conscious cognition associated with low-intensity stimuli); and Associative Sensitivity (i.e., Spontaneous cognitive content that is not related to standard associations with the environment).

The scoring process is the same for all scales, and a higher score on one scale means that temperament is more saturated on that scale.

In the present study, a Portuguese Version of the ATQ was used. As in the original version, this version presents four factorial scales. All dimensions have a good consistency with *Alfa Cronbach* from 0,87 in the Effortful Control subscale to 0,92 in the Negative Affect [44].

### Assessment of oxytocin serum levels

Blood samples were collected between 8 and 8:30 a.m. after 12 h of fasting and between 5 and 8 day of the menstrual cycle to avoid bias since some previous research suggests fluctuation of oxytocin levels during the circadian phases and throughout the menstrual cycle [45]**.** Blood was centrifuged at 1500×*g* for 15 min, after which serum was separated and stored at − 80 °C until the analysis.

Samples were extracted and oxytocin concentration were quantified in duplicates using a commercial enzyme-linked immunosorbent assay (ELISA, Abcam ab133050). This Kit show a intra-assay CV < 13.3% and inter-assay < 20.9%, sensivity 15 pg/ml. According to the recommended kit ELISA method, a solid-phase extraction of the serum samples was performed to eliminate the effects of potentially interacting molecules such as vasopressin [46]. A volume of 125 μL of 0.1% trifluoracetic acid in water (TFA-H2O) was added to a same volume of serum sample and centrifuged at 17,000×*g* for 15 min at 4 °C and the supernatant was collected. The C18 Sep-Pak column (Sep-Pak VAC, C18 3 cc/200 mg, Waters) was equilibrated with 1 mL of acetonitrile and then four times with 3 mL of 0.1% TFA-H2O. The supernatant was applied to the C18 Sep-Pak column and washed four times, with 3 mL of 0.1% TFA-H2O; the eluted fraction was discarded. The sample was recovered by applying a 3 mL solution of 60% acetonitrile and 40% 0.1% TFA-H2O. Next, the solvent was evaporated at 4 °C using a vacuum centrifugal concentrator. Samples were resuspended in the appropriate sample diluent solution supplied in the kit, and oxytocin levels were assessed using the corresponded standard competitive ELISA method.

### Statistical analysis

Data were analyzed using the Statistical Software SPSS, version 26.0 for Windows.

Shapiro–Wilk test was used to test data normality. Results were considered significant at a 5% significance level.

A descriptive univariate analysis, including central tendency and dispersion, was performed for all quantitative variables (i.e., OT; BDI-II; ATQ) and frequency analysis (n, %) for qualitative data. The one-way analysis of variance (ANOVA) was used to determine statistically significant differences between groups.

Bivariate association between oxytocin serum levels, BDI-II sores and ATQ (temperament dimensions: Negative Affect, Extroversion, Effort Control and Guiding Sensitivity) was assessed using Pearson's correlation coefficient.

A Hierarchical multiple regression analysis was performed to identify depression predictors. In this model Negative affect scores, Effort control scores, Activation control scores and Oxytocin serum levels were considered independent variables. The obtained model obeys the Gauss-Markov conditions (zero mean residuals, normal residuals, and residuals with constant variance).

## Results

### Association between plasma oxytocin serum levels and depression

Forty-five women aged between 18 and 25 years old (19.37 ± 1.32 years) volunteered to participate in this study. A total of 9 (20%) received psychological support at that moment, but all participants were free of psychotropic medication for at least three months.

Oxytocin serum levels ranged from 14.42 to 265.03 pg/mL, and the mean value was 122.44 pg/mL (SD 59.67). The mean score of Depression (BD-II) was 15.20 (SD 6.68), ranged between 2 and 37, 40% (n = 18) of participants show no clinical depressive levels; 42.2%(n = 19) showed mild levels; 15.6% (n = 7) showed moderate levels; and 2.2% (n = 1) showed severe levels.

**Fig. 1:**
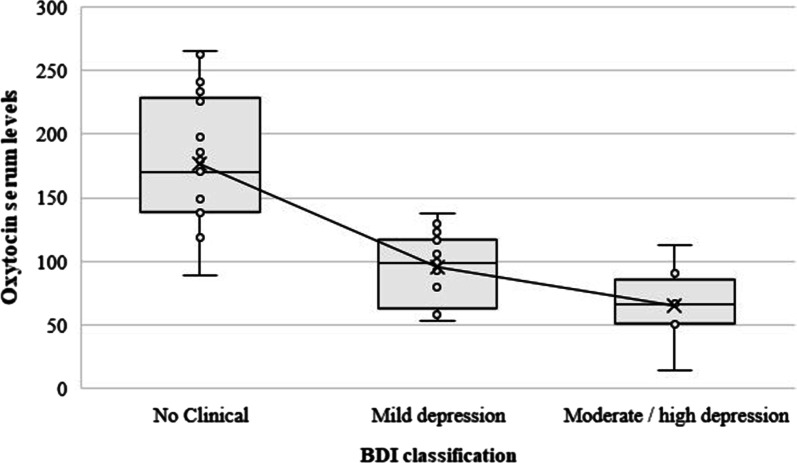
Oxytocin (pg/mL) and depression levels: The solid line connects the x symbols (marginal means). The dots within the box-plot represent the observed serum values of oxytocin. There were no "outliers"

**Fig. 2:**
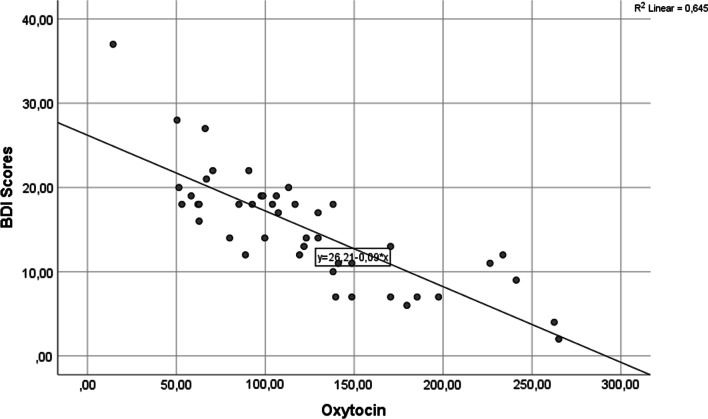
Sactterdot for oxytocin (pg/mL) versus BDI scores

Oxytocin serum levels were significantly different among groups, being lower in moderate and severe depression (*p* < 0.0001) (Fig. 1). The oxytocin serum levels were strong and negatively correlated with BDI score (r = − 0.803; *p* < 0.0001) (Fig. 2).

### Association between OT serum levels and temperament dimensions

The strongest dimension of Temperament found in participants was “Negative affect” (n = 29, 64.4%) followed by "Orienting sensitivity" (n = 23, 51.1%), “Extroversion” (n = 15, 33.3%) and “Effortful control” (n = 2, 4.4%).

The oxytocin levels were moderately and negatively correlated with "Negative affect" (r = − 0.586; *p* < 0.0001). A moderate positive correlation was found between oxytocin serum levels and "Effortful control" (r = 0.541; *p* < 0.0001) and "Activation control" (r = 0.360; *p* = 0.015), meaning that lower levels of oxytocin were related with lower "Effort control" and "Activation control". No significant correlations were found between the remaining temperament dimensions and oxytocin serum levels.

### Regression model

A hierarchical multiple regression model using a stepwise method was conducted to identify predictors of depression. In the first step, the four subscales of temperament were considered as regressors. Only "negative affect" was a significant predictor (B = 4.182, *p* = 0.001). This model presents an Adjusted R^2^ equal to 0.211 (R^2^ Change = 0.229, F Change (1,43) = 12.755, *p* = 0.001), explaining 21.1% of the depression variation, verifying that for each additional value in this subscale, on average the depression score increases by 4.182.

In a second step the four subscales of temperament and oxytocin serum levels were considered as regressors. The only predictor found was oxytocin (B = − 0.090, *p* < 0.0001). This model presents an Adjusted R^2^ equal to 0.637 (R^2^ Change = 0.645, F Change(1, 43) = 78.100, *p* < 0.0001), explaining 63.7% of the depression variation. This means that for each extra value in oxytocin levels, a decrease of 0.09 in the depression score, on average. The inclusion of "oxytocin plasma level" in the model annulled the effect of "negative affect" temperament dimension on depression.

### Mediation model

A new model was built to identify a possible mediation role of oxytocin in the relationship of "Negative affects" and depression (BDI). The oxytocin levels were included as a dependent variable and the temperament subscales as independent variables. In this model, negative affects (B = − 40.67, *p* = 0.000) and effort control (B = 36.75, *p* = 0.001) were found as regressors of oxytocin, meaning that for each additional unit in negative affects, there is an average decrease of 40.67 pg/mL. For each unit added in the control of exhaustion, an increase of 36.75 pg/mL in oxytocin values occurs on average. This model presents an Adjusted R^2^ equal to 0.556 (R^2^ Change = 0.115, F Change(1, 47) = 12.686, *p* = 0.001), which explains 56.6% of the oxytocin variation.

**Fig. 3:**
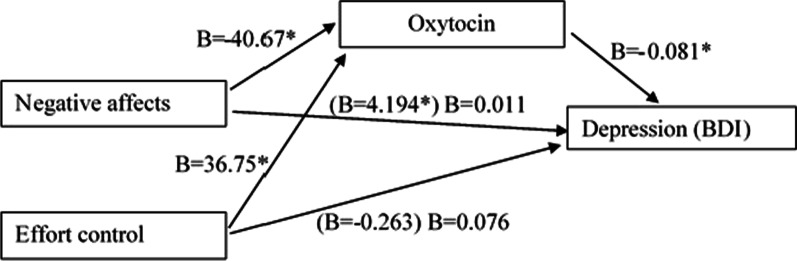
Effect of oxytocin mediation between negative affects and effort control for depression (BDI). (B)—Coefficients of the multiple regression model only with the temperament dimension

Despite de need for futher research, these results showed that the relationship between "Negative affects" and depression (BDI) can be partially mediated by oxytocin (Fig. 3).

## Discussion

The present study aimed to assess the relationship between serum oxytocin, depression and temperament traits and to analyze the effect of serum oxytocin and temperament in depressive symptomatology in a sample of 45 young, healthy university female students.

### Prevalence of depression

The results show that more than half of the participants report mild or moderate symptoms of depression. These results are higher than the mean prevalence of depressive symptomatology reported in the systematic review by Ibrahim et al. [5] and are also higher than the one found in Portuguese female college students by Campos and Gonçalves [43]. These results are consistent with some studies pointing to an increase in depressive symptomatology among young Portuguese women [47] and reinforce the idea that a significant number of Portuguese university females live with depressive symptomatology [5].

### Association between depression symptoms and oxytocin levels

In our study, OT serum levels were strong and negatively associated with depression scores suggesting that lower levels of OT are associated with higher levels of depression. Despite some mixed findings, in previous studies, a negative correlation between OT and depression has also been reported in clinical and non-clinical populations. For example Scantamburlo et al. [27] found a negative correlation between depressive symptoms and serum OT concentration in a clinal sample of major depressed adults. Supporting these results, Ozsoy et al. [29] also reported decreased serum OT levels in inpatients with major depressive disorder compared with healthy controls. In that study female patients showed lower levels than controls, while no difference was seen between the male groups. Further, Gordon et al. [28] found an inverse correlation between psychological distress, particularly depressive symptoms, and plasms OT in a sample of healthy university students.

Other studies showed inconsistent results [48] or, as in a systematic review by Engel et al. [35], found, in geneal, no significant differences in basal endogenous oxytocin concentrations between depressive patients and healthy controls. Nonetheless, in that systematic review, the authors pointed out that the effect was heterogeneous and that results varied concerning participants (patients and control groups), clinical and demographic characteristics, and methodological designs. Indeed, there are some differences regarding the samples demographic characteristics, between the studies included in the meta-analysis and the one used in our study. Unlike our study, the meta-analysis includes studies with adults, men and women, the mean age varying between 31 and 48 years between studies. Further all studies in the meta-analysis examined a currently depressed patient, with five studies reporting a patient with current medication. Despite other reasons that should be addressed in future studies, these differences may account for the non-similarity of the results.

### Association between temperament dimensions and oxytocin levels

Regarding the association between temperament dimensions and OT levels, our study showed a negative correlation with "Negative affect" and a positive correlation with "Effort Control”. These results suggest that lower levels of OT are associated with temperamental traces of higher vulnerability to distress, lower mood and less energy to cope with suffering and loss. On the other hand, higher OT levels were associated with the personal capacity to self-regulation and to engage in active coping. Our results are consistent with the findings of the few existing studies. Bell et al. [49], in a study with major depressive episode patients, found a positive association between OT levels and the personality dimensions of "Reward dependence", linked with noradrenergic activity and defined as the tendency to respond to reward signals that maintain behavior; and "Novelty seeking", defined as the tendency of frequent activation or initiation of behaviors in response to novel stimuli and associated with the neurotransmitter dopamine. In the same line, De Dreu et al. [50], in a study with university students, reported that "Novelty-seeking" was positively associated with plasma oxytocin. Similar, Andari et al. [51] found a positive correlation between oxytocin plasma levels and extroversion, in healthy male and female adults. Further, in a population of medication free psychiatric patient’s plasma oxytocin levels showed a significant positive correlation with Impulsiveness factor (Impulsiveness, Monotony Avoidance) [52]. These results reinforce the idea that OT plays an important role in human prosocial, proactive and towards novel behavior orientation.

"Negative affect" was only independent a predictor of depression. In the final regression model, the predictive value of this temperament dimension decreased when OT plasma levels entered the model. In fact OT level was the main predictor of depression. In our study OT was found to have a mediation role between "Negative affect" and depression. More significant "Negative affects" and lower values of oxytocin lead to higher levels of depression the results support the hypothesis of oxytocin as a biomarker of emotional changes that characterize depression, namely "Negative affects" [32]. Although this is explanatory study, the role of OI as meditar and/or a moderator in emotional and social functions, as already been highlight in previous studies [53] and this finding should be addressed in further research.

A possible limitation of our study is related to the ELISA kit choice to assess the oxytocin levels (Abcam ab133050). The Elisa kits from others companies show considerable different serum oxytocin concentrations. We observed that kit Abcam presents higher values than the others. However, we consider that this does not compromise our objectives or conclusions. Although we could consider a limitation of our study the absence of evaluating oxytocin levels within the Central Nervous System (CNS), several authors show a strong relationship between CNS oxytocin levels and its peripheral circulation levels [54, 55].

Our results should be read with caution due to the characteristics of the sample essentially the prevalence of depression a and the low numbers of subjects scoring above cutoff for “Effortful control”. Furthermore the small number of participants advises restrict conclusions.

In resume, our results showed that OT level, rather than personality dimensions, was associated with depressive symptomatology. These results highlight the importance of studying the role of oxytocin in human emotional and social process, and the links between OT and other physiological and neurocognitive variables (e.g. information process; perception of emotional stimulus).

## Data Availability

The datasets used and analyzed during the current study are available from the corresponding author on reasonable request.
